# Positive Surgical Margins in the 10 Most Common Solid Cancers

**DOI:** 10.1038/s41598-018-23403-5

**Published:** 2018-04-09

**Authors:** Ryan K. Orosco, Viridiana J. Tapia, Joseph A. Califano, Bryan Clary, Ezra E. W. Cohen, Christopher Kane, Scott M. Lippman, Karen Messer, Alfredo Molinolo, James D. Murphy, John Pang, Assuntina Sacco, Kathryn R. Tringale, Anne Wallace, Quyen T. Nguyen

**Affiliations:** 1 Division of Otolaryngology, Head and Neck Surgery, University of California, San Diego, CA USA; 2 Department of Surgery, University of California, San Diego, CA USA; 3 Department of Pharmacology, University of California, San Diego, CA USA; 4 Moores Cancer Center, University of California, San Diego, CA USA; 5 Department of Medicine, Division of Hematology-Oncology, University of California, San Diego, CA USA; 6 Department of Urology, University of California, San Diego, CA USA; 7 Department of Radiation Medicine and Applied Sciences, San Diego, CA USA

## Abstract

A positive surgical margin (PSM) following cancer resection oftentimes necessitates adjuvant treatments and carries significant financial and prognostic implications. We sought to compare PSM rates for the ten most common solid cancers in the United States, and to assess trends over time. Over 10 million patients were identified in the National Cancer Data Base from 1998–2012, and 6.5 million had surgical margin data. PSM rates were compared between two time periods, 1998–2002 and 2008–2012. PSM was positively correlated with tumor category and grade. Ovarian and prostate cancers had the highest PSM prevalence in women and men, respectively. The highest PSM rates for cancers affecting both genders were seen for oral cavity tumors. PSM rates for breast cancer and lung and bronchus cancer in both men and women declined over the study period. PSM increases were seen for bladder, colon and rectum, and kidney and renal pelvis cancers. This large-scale analysis appraises the magnitude of PSM in the United States in order to focus future efforts on improving oncologic surgical care with the goal of optimizing value and improving patient outcomes.

## Introduction

One in four deaths in the United States (US) is due to cancer^1^. Treatment modalities vary considerably depending on stage and location, however surgical excision is an integral part of treatment for most solid tumors. The goal of surgical resection is the eradication of cancer-–both gross and microscopic. A positive surgical margin (PSM) occurs when this ideal is not achieved, and cancer cells are present at the edge of the resection specimen. The cancer biology, responses to neo-adjuvant and adjuvant therapies, and treatment paradigm differs across tumor types. Similarly, the impact that a PSM has on prognosis and treatment decisions depends on tumor type. For example, the 2017 National Comprehensive Cancer Network (NCCN) guidelines for kidney and ovarian cancer do not include surgical margins, but margins are mentioned 16 times in the oral cavity cancer guidelines. In general, PSMs warrant additional (adjuvant) treatments, which confer significant increased costs and burden to the patient and healthcare system^2^.

To broadly characterize the scope of PSMs in surgical cancer care, we used today’s largest oncology database, the National Cancer Data Base (NCDB), to evaluate PSM prevalence in the ten most common solid organ cancers in the US.

## Methods

The ten most common solid organ cancers in the US are: prostate, breast, lung and bronchus, colon and rectal, urinary bladder, thyroid, kidney and renal pelvis, uterine corpus, oral cavity, ovarian^3^. We included NCDB data (1998–2012) of patients with these cancers as their only malignancy (n = 10,400,589 Supplemental Table 1), and excluded patients: treated without surgery (n = 3,028,552), who underwent local tumor destruction (n = 98,450), with unknown surgery status (n = 62,071). Of the remaining 7,211,516 patients who underwent surgical resection of their cancer, surgical margin information was available for 6,495,889 (90.1%, Supplemental Table 1).

We examined the NCDB variable “margin status”, which represents presence or absence of tumor following primary resection. PSM was defined as “microscopic residual tumor” (n = 311,635), “macroscopic residual tumor” (n = 48,871,) or “residual tumor, not otherwise specified (NOS)” (n = 214,675). Negative margins were defined as “no residual tumor” (n = 5,920,708). For patients undergoing multiple sequential surgeries, the NCDB only reports the margin status of the final procedure, but does not specify which patients had multiple surgeries. Tumor (T) categories in the NCDB are based on the American Joint Committee on Cancer (AJCC) 6th and 7th editions.

PSM rates were determined for each cancer site by year, T category, gender, age group, and race. To assess PSM change over the study period, we compared PSM rates for the last 5 years (2008–2012) to the first 5 years (1998–2002), using the two-proportion z-test with pooled standard error. Additionally, we conducted a multivariable logistic regression to investigate the adjusted effect of these time periods on PSM. Patient-related covariates included: gender, age, race/ethnicity, income, Charlson-Deyo^4^ comorbidity index (CI), and insurance type. Median household income, estimated by correlating postal code time of diagnosis with American Community Survey data, 2008–2012 and adjusted for inflation, was categorized by quartile (1st quartile < $38,000; 2nd $38,000–47,999; 3rd $48,000–62,999; 4th > $63,000). Tumor-related covariates included: tumor stage (T-stage) and histologic grade (American Joint Committee on Cancer (AJCC) seventh edition guidelines for pathologic staging5). Healthcare system covariates included: hospital tumor-specific case volume, facility type, and geographic location. Hospital tumor-specific volume was calculated using average number of cases reported to the NCDB per year (low volume < 25th percentile, high volume > 75th percentile). Facility type was based on CoC accreditation criteria. Statistical analyses were performed using STATA, version 13 (Stata, StataCorp LP, College Station, Texas).

The prognostic implications of PSM and accompanying adjuvant treatment considerations and costs were summarized. Adjuvant treatment recommendations were obtained from National Comprehensive Cancer Network (NCCN). This study was approved by the UCSD Institutional Review Board (protocol #150107). All methods were performed in accordance with relevant guidelines and regulations.

### Disclosure

The American College of Surgeons and the CoC have not verified and are not responsible for the analytic or statistical methodology employed, or the conclusions drawn from these data by the investigator.

## Results and Discussion

Most patients were women (66.2%) and White (76.04%). There were 2,530,565 breast, 761,637 prostate, 241,791 bladder, 1,218,834 colon and rectum, 314,459 thyroid, 120,826 oral cavity, 462,282 lung and bronchus, 361,240 kidney and renal pelvis, 391,997 uterine, and 92,058 ovarian cases (Supplemental Table 1). There were 9.38% *in situ*, 43.25% T1, 20.04% T2, 13.74% T3, and 3.99% T4 cases. For each cancer site, PSM increased with higher tumor category and grade (Table 1).

**Table 1:** Prevalence of PSM for individual tumor sites as a function of gender, race, age, tumor category, tumor grade. Analysis of PSM change over study period comparing unadjusted rate from the last 5 years of the study period 2008–2012 to the first 5 years of the study period (1998–2002).

	BREAST (LUMP, MAST, TOTAL)			PROSTATE	BLADDER	COLON & RECTUM	THYROID	ORAL CAVITY	LUNG & BRONCHUS	KIDNEY & RENAL PELVIS	UTERINE	OVARIAN	TOTAL
**Number of Cases (n)**	1,479,230	1,051,335	2,530,565	761,637	241,791	1,218,834	314,459	120,826	462,482	361,240	391,997	92,058	6,495,889
Number of PSM Cases	97,335	55,746	153,081	160,194	23,317	83,241	36,230	15,411	33,861	20,691	16,938	32,217	575,181
Overall PSM Rate (%)	6.58	5.30	6.05	21.03	9.64	6.83	11.52	12.75	7.32	5.73	4.32	35.00	8.85%
**Gender**													
Men	8.30	4.47	5.53	21.03	8.96	6.71	13.24	12.65	8.00	6.04	N/A	N/A	7.73% (109,572)a
Women	6.57	5.32	6.05	N/A	11.58	6.95	10.99	12.92	6.64	5.23	4.32	35.00	6.69% (256,260)a
**Race**													
White	6.32	5.08	5.81	20.60	9.31	6.66	10.90	12.30	7.15	5.80	3.86	35.67	8.60% (424,725)
Black/AA	7.93	6.53	7.32	23.19	14.19	7.43	8.76	18.62	8.53	4.99	7.90	33.47	10.04% (58,393)
Other	7.28	5.68	6.57	22.21	9.20	7.12	15.32	13.62	7.46	5.82	4.78	27.81	9.34% (19,087)
Latino/Hisp	7.07	5.53	6.41	21.65	10.28	7.34	14.53	13.15	7.83	5.84	4.66	33.86	9.47% (72,976)
**Age**													
<40	8.62	6.32	7.33	17.17	6.94	9.78	10.43	10.56	9.56	4.20	3.52	15.64	8.42% (28,867)
41–80	6.15	5.12	5.73	21.08	8.78	6.75	11.60	12.70	7.25	5.72	4.16	36.49	8.89% (494,280)
>81	10.18	6.14	8.45	17.07	13.41	6.68	23.78	14.09	7.79	7.10	6.78	42.76	8.74% (52,034)
**T-category**													
*In Situ*	5.67	3.19	4.89	0.00	3.30	1.41	0.00	9.33	4.80	3.26	0.58	N/A	4.39% (26,766)
T1	5.63	3.74	4.99	19.02	6.24	2.74	5.21	7.85	3.05	2.87	1.00	3.94	5.96% (167,291)
T2	9.13	5.27	6.97	19.58	17.37	1.92	8.26	14.92	6.77	2.73	5.87	27.78	8.50% (110,683)
T3	20.11	9.55	11.05	45.95	24.07	5.61	24.79	20.32	19.57	14.17	25.70	58.25	11.26% (100,539)
T4	37.60	18.58	20.89	65.54	40.33	27.05	50.76	25.18	27.13	42.04	51.89	N/A	29.96% (77,740)
**Grade**													
Well Differentiated	5.19	4.14	4.85	9.64	2.41	4.68	9.10	9.70	4.45	2.73	0.02	10.35	4.47% (44,669)
Mod Differentiated	6.66	5.23	6.07	15.99	4.75	5.82	19.52	13.81	6.32	3.61	2.53	25.98	7.51% (195,187)
Poorly Differentiated	7.21	5.94	6.59	27.31	14.47	12.47	32.77	18.61	8.71	8.44	10.77	45.22	13.11% (229,211)
Undifferentiated	8.45	6.95	7.76	31.51	16.26	15.54	61.89	20.57	9.91	16.28	15.58	48.31	16.46% (24,681)
**Change over study period**													
Men			−3.22** (**−**3.96:**−**2.47)	**−0.213** (**−**0.44:0.02)	2.95** (2.62:3.27)	2.41** (2.25:2.56)	−0.775* (**−**1.43:**−**0.12)	**−0.252** (**−**0.83:0.33)	−0.374* (**−**0.65:**−**0.10)	1.44** (1.18–1.70)	NA	NA	
Women			−3.63** (**−**3.70:**−**3.55)	NA	3.72** (3.11:4.33)	2.38** (2.23:2.54)	0.528* (**−**0.12:0.19)	−1.15*v(**−**1.89:**−**0.41)	−0.74** (**−**0.99:**−**0.49)	1.19** (0.88:1.49)	0.481** (0.33–0.64)	−4.59** (**−**5.2:**−**3.97)	

a. Only tumors occurring in both genders are included here.

b. Numbers given are percents unless otherwise indicated. *p < 0.05; **p < 0.001, Not applicable (NA).

### PSM trends over time

**Figure 1:**
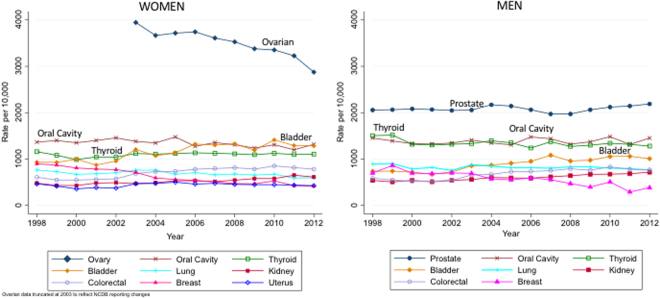
Positive surgical margin (PSM) prevalence (reported as rate per 10,000 patients) for each cancer in women (**A**) and men (**B**) as a function of time.

Unadjusted PSM rates for breast, and lung and bronchus cancer in both men and women declined over the period of the study (Fig. 1A,B, Table 1). In contrast, PSM rates for bladder, colon and rectum, and kidney and renal pelvis increased for both genders. Ovarian cancer PSM rates decreased over time (Fig. 1A,B, Table 1).

Multivariable logistic regression analysis showed that patients treated in the second seven years (2006–2012) were more likely to have PSM for bladder (OR 1.09, p = 0.007) and kidney (OR 1.29, p < 0.0001) tumors, and less likely to have PSM for breast (OR 0.78, p < 0.0001), lung (OR 0.93, p = 0.002), and prostate (OR 0.96, p = 0.045) tumors (1998–2005) (Table 1). This means that a patient having surgery between 2006 and 2012 had a 22% lower chance of having a PSM than a patient treated during the 1998–2002 time period.

### Clinical and financial impact PSM

The prognostic implications of PSM and accompanying adjuvant treatment considerations and costs are summarized in Table 2 for eight of the tumor sites—breast, prostate, bladder, colon and rectum, thyroid, oral cavity, lung and bronchus, uterine. The NCCN treatment guidelines for kidney and renal pelvis and ovarian cancers do not account for PSM, but a summary of corresponding PSM implications is provided in the manuscript text.

**Table 2:** Summary of the prognostic implications, adjuvant treatment recommendations, and associated cost estimates of PSM for individual tumor sites: breast, prostate, bladder, colon and rectum, thyroid, oral cavity, lung and bronchus, uterine.

		BREAST	PROSTATE	BLADDER	COLON & RECTUM
Prognostic Implications for PSM		Increased LRR, decreased DSS	Increased risk of biochemical recurrence; no impact on cancer-specific mortality	Increased LRR, Decreased DSS	Increased LRR and DM, decreased DFS and OS
Adjuvant Treatment Recommendations for PSM based on NCCN Guidelines	Early Stage	PSM after lumpectomy: Re-excision OR Mastectomy OR Higher RT boost for focal PSM without extensive intraductal component	RT	Stage II (T2N0) RT, OR Chemo (if no neoadjuvant therapy) Stage II (T2N0) primary *urethra carcinoma* (males): Re-resection Or CRT (preferred) Or RT	Colon: larger resection, T3N0M0 chemo, and for any stage consider boost radiation
	Late Stage	PSM after mastectomy: Consider RT to chest wall +/− infraclavicular region +/− supraclavicular area +/− internal mammary nodes &any part of axillary bed at risk	RT	Stage III-IV (T3–4, N0-2) urothelial: Higher RT boost	Rectum: larger resection, T1Nx after trans-anal excision: Transabdominal resection +/− CRT or CRT, and intra-operative radiation therapy (IORT) may be considered for PSM especially in T4 and recurrent cancers
Cost of Adjuvant Treatment due to PSM	Surgery	Lumpectomy alone: $70,520 Mastectomy alone: $48,258 Mastectomy + Recon: $88,089			
	Radiation	RT: $8,600	RT: $18,000		
	Chemotherapy			Chemo: $16,416	Chemo: $17,833
		**THYROID**	**ORAL CAVITY**	**LUNG & BRONCHUS**	**UTERINE**
Prognostic Implications for PSM		No impact	Increased LRR, Decreased OS	Decreased OS	Potential increase in LRR
Adjuvant Treatment Recommendations for PSM based on NCCN guidelines	Early Stage	PSM after partial thyroidectomy (papillary): Completion thyroidectomy	STAGE I-II: Re-resection *OR* RT *OR* CRT (T2 only)	STAGE IA: Re-resection (preferred) *OR* RT (category 2B) STAGE IB-IIA (node-negative): Re-resection (preferred) +/− chemo *OR* RT +/− chemo (chemo for stage IIA) STAGE IIA-B (node-positive): Re-resection+ chemo *OR* CRT	Vaginal brachytherapy and/or RT for stage I after extrafascial hysterectomy
	Late Stage		STAGE III-IV CRT (category 1) *OR* Re-resection *OR* RT	STAGE IIIA: CRT	T1, Nx rectal after trans-anal excision: Transabdominal resection +/− CRT OR CRT
Cost of Adjuvant Treatment due to PSM	Surgery	Surgery: $5,617	Surgery: $24,595	Surgery: $15,034	
	Radiation		RT: $21,300	RT: $9,000	RT: $9066 Brachytherapy: $7233
	Chemotherapy		Chemo + RT: $27,928		

^*^Unless otherwise specified, recommendations are category 2 A (uniform NCCN consensus that intervention is appropriate based on lower-level evidence). Kidney and Ovarian excluded as surgical margin status does not affect adjuvant therapy recommendations.

^**^Cost for adjuvant treatment was obtained from published data in the United States (listed in U.S. dollars) where available.

NCCN: National Comprehensive Cancer Network, PSM: positive surgical margin, RT: radiation therapy; chemo: chemotherapy, CRT: chemoradiation; LRR: locoregional recurrence rate, DSS: disease-specific survival, DM: distant metastasis, DFS: disease-specific survival, OS: overall survival.

### PSM for cancers affecting both genders

The highest PSM rates for cancers affecting both genders were oral cavity (12.75%), followed by thyroid cancers (11.52%). The greatest gender-specific difference in PSM rate was seen in bladder cancer (11.58% in women vs. 8.96% in men).

#### Breast cancer

Lumpectomy and mastectomy PSM rates were 6.58% and 5.30%, respectively (Table 1). PSM rates for lumpectomy is significantly lower than the reported national estimate of 22.9%^5^. This is likely due to NCDB’s practice of reporting final PSM, even if a patient has had multiple sequential procedures. Breast cancer patients may undergo multiple sequential lumpectomies, followed by mastectomy for persistent PSM.

Following mastectomy, PSM in node-negative disease is associated with an increased locoregional recurrence (LRR) and decreased disease-specific survival (DSS) (Table 2)^6^. In a meta-analysis, Moran *et al*. found that patients with close or PSM had 2-times greater odds of developing tumor recurrence than patients with negative margins^7^.

NCCN guidelines^2^ for patients with PSM is stratified by stage (Table 2). Patients with early-stage tumors are recommended to undergo re-excision, mastectomy; or for focal PSM without extensive intraductal component, a high chest wall irradiation boost. One in four women who undergo an attempt at breast-conserving therapy have positive or unclear margins and go on to have re-excision^5,8^. For patients with PSM after mastectomy, the NCCN recommends radiation to the chest wall, infraclavicular and supraclavicular regions, and potentially to the internal mammary nodes and any other part of the axillary bed at risk. These additional procedures confer increased discomfort and stress, increased risk of complications, potentially compromised aesthetic outcomes, and increased health care costs for both the patient and the healthcare system^7,9–11^.

#### Oral Cavity cancer

The PSM rate for oral cavity cancer was 12.75% (Table 1)—the highest overall PSM rate for tumors affecting both genders. This finding is consistent with prior figures, although it is important to note that previously reports on PSM rates ranged widely (range 1–22%) and with some exceptions^12^, were derived primarily from single-institution studies, and often included extra-oral sites^13–16^.

The prognostic implication of PSM in oral cavity cancer is significant. PSMs are independently associated with increased risk of LRR and decreased overall survival (OS) (Table 2)^13–16^. Sutton *et al*. reported a relative risk of death of 11.61 (p = 0.0013) for patients with PSM and 2.66 (p = 0.02) for patients with close margins, compared to those with negative margins^17^.

NCCN^2^ recommends that oral cavity cancer patients with PSM receive adjuvant therapy (Table 2), which is stratified based on stage. Early-stage patients (stage I-II) should undergo re-resection to obtain negative margins when feasible; otherwise they should receive radiation, or radiation with chemotherapy (for T2 tumors only). Locally advanced stage patients (stage III-IV, non-metastatic) should receive radiation with chemotherapy (category 1 recommendation), re-resection, or radiation alone. The need for adjuvant therapy–whether it be surgery, radiation, or radiation with chemotherapy–confers increased healthcare costs (Table 2) and subjects the patient to additional toxicities which can adversely impact quality of life^18^.

#### Bladder

PSM rate for bladder cancer was 9.64%, with women having higher PSM (11.58%) compared to men (8.56%) (Table 1). Our study is limited by NCDB coding which does not differentiate transurethral resection of bladder tumors from radical cystectomies. Thus, the PSM rates reported here reflect a combination of these two different procedures. Published PSM rates are divided based on presence or absence of muscle invasion, where non-muscle invasive refers to Ta, CIS or T1 tumor category, and muscle-invasive refers to all other tumor categories^2^. Based on a comprehensive literature review, Divrik *et al*. reported that the rates of residual tumor in non-muscle invasive bladder cancer after a transurethral resection of the bladder ranged from 28% to 74%^19^. In contrast, our study finds that non-muscle invasive tumors (Ta, CIS, T1) had a PSM rate of 6.24% at radical cystectomy. For muscle-invasive bladder cancer, a 2010 multi-site study of 513 patients reported a 6.8% rate of PSM^20^, which is much lower than our PSM rates ranging from 17.37–40.33% (T-categories 2–4). This variability is likely due in part to differences in the underlying characteristics of the study populations, and to the inclusion of low and high volume centers in the NCDB.

Incomplete resection of bladder tumor, irrespective of muscle invasion has been associated with unfavorable prognosis (Table 2). Studies of PSM in patients with non-muscle invasive cancer showed decreased recurrence-free survival. Dotan *et al*. examined the importance of margins in patients undergoing radical cystectomy for muscle-invasive bladder cancer. They demonstrated that PSM had a profoundly negative impact on cancer specific survival, which decreased from 72% to 32% at 5 years. They also demonstrated that patients with PSM had a 3.5-fold increased risk (21% vs 6%) of local recurrence at 5 years, which is almost uniformly fatal. On multivariate analysis, PSM was an independent predictor of death from cancer^21^. The higher PSM rate for women may be due to the increased technical challenge of radical cystectomy and anterior exenteration in women which requires anterior vaginal dissection and hysterectomy and salpingo-oophorectomy.

NCCN^2^ recommends adjuvant radiotherapy or chemotherapy for patients with stage II margin positive disease (Table 2). Radical cystectomy is relatively contraindicated in patients with positive urethral margin, and chemoradiation is preferred^22^. Late stage bladder cancer requires higher radiotherapy boost^2^.

#### Colon and Rectum

The total PSM rate for colon and rectal cancer was 6.83%. Higher PSM rates correlated with higher T-category (Table 1), a finding consistent with prior reports^23^. For colon cancer, PSM is a poor prognostic feature for T2 and T3 tumors^24^. For rectal cancer, PSMs are considered a high-risk pathologic feature. In a Dutch study on rectal cancer, patients with PSM had a local recurrence rate of 22%, compared with 4% of those with negative margins^25^. Lin *et al*. also showed that when defining a PSM as less than 1 mm from the tumor, the rate of distant metastasis was 61.5%, compared with 15.2% for margins of >1 mm, making PSM a predictor for the development of distant metastasis^26^ and decreased disease-free survival^27^.

Based on NCCN guidelines^2^, PSM in colon cancer may warrant larger resection, chemotherapy, and consideration for boost radiation (Table 2)^2,27,28^. For rectal cancers, PSM may mean larger resection with or without chemoradiation, and intra-operative radiation therapy (IORT) may be considered for PSM especially in T4 and recurrent cancers.

#### Kidney and Renal Pelvis

In this study, the PSM rate for patients with kidney and renal pelvis cancer was 5.73% (Table 1). Our data are similar to the literature review by Borghesi *et al*. in 2013, reporting that the overall incidence of PSMs after nephron-sparing surgery (NSS) when performed electively is quite low, ranging from 0% to 7%^29^. Comparable PSM rates have been reported between different surgical approaches: 0% to 7% in open partial nephrectomy, 0.7% to 4% in laparoscopic partial nephrectomy (LPN), and 0.7% to 4% in robot-assisted partial nephrectomy^30^. Patients with an imperative indication for NSS seem to have a higher risk of PSM incidence (8.9–27.5%), likely due to the presence of larger tumors or unfavorable tumor location^29,31^.

There is no clear consensus on clinical implications of PSM in these cancers. Some authors have found limited influence on long-term oncological outcomes^32^, whereas others showed that PSM increased the hazard ratio of recurrence and metastasis^33^. Borghese *et al*. also reported that local recurrence seems to be more likely in patients with PSMs, especially in those with high-grade tumors^29^. NCCN guidelines for kidney and renal pelvis cancers do not account for PSM^2^.

#### Lung and Bronchus

We found an overall PSM rate of 7.32% for lung and bronchus cancers (Table 1), consistent with prior reports of PSM rates ranging from 6–7.8%^34,35^. Interestingly, we found higher PSM rates in young patients (<40 years). Previous work has shown that among young patients, male sex, non-adenocarcinoma histology, black race, and main bronchial primary site are independent negative prognostic factors^36^.

Surgical resection plays a critical role in the treatment of lung cancer^37^, often providing the only potentially curative treatment for non-small cell lung cancer (NSCLC). However, clear surgical margins are difficult to obtain due to the limited amount of resectable tissue. Furthermore, the presence of occult micro-metastases are not uncommon^38^. Five-year OS following surgery for patients with stage I-III NSCLC is less than 50%^39,40^ and 30–55% of NSCLC patients who undergo curative resection develop recurrence and die of their disease^38^. PSMs are associated with poor prognosis, significantly impact survival irrespective of stage^37^ and approximately halve the five-year survival rate^41–43^. Moving forward, tumor markers and intraoperative visualization may help better establish early- versus advanced-stage disease^44–46^.

NCCN (Table 2)^2^ recommends adjuvant treatment for PSMs based on stage^11^. Re-resection is typically preferred for early-stages with the option of concurrent chemotherapy, and alternatively, radiotherapy. For later stages, chemoradiotherapy is recommended as the preferred treatment in cases of PSM, which confers additional costs (Table 2).

#### Thyroid

Thyroid had one of the highest PSM rates (11.52%) of all cancers in this study (Table 1). This is comparable to a report of 10% PSMs from a retrospective study of patients with differentiated thyroid cancer^47^. Over the study period, the prevalence of PSM rates decreased in men, yet increased in women. This is interesting, particularly given less aggressive histologic subtypes are more common in women^48^. The highest PSM rate was seen in the elderly age group (23.78%).

Management of thyroid cancer is controversial due to the absence of high-level evidence regarding resection margins or adjuvant radiation therapy^47,49^. A large NCDB study showed that total thyroidectomy results in lower recurrence rates and higher survival for papillary thyroid cancer, when compared to lobectomy^50^. However, a more recent NCDB study did not observe a survival advantage with more extensive surgery^51^. Incomplete tumor resection has been recognized as one of the important poor prognostic factors in thyroid cancer patients who undergo total thyroidectomy^52^. American Thyroid Association has classified incomplete tumor resection as group at high-risk of recurrence^53^. In patients with non-invasive thyroid cancer, prior work has shown a significantly higher rate of early recurrence in the PSM group than the negative margin group—but all early recurrences were in regional lymph nodes of surgically non-dissected neck areas^52^. Disease-free survival has been shown to be impacted by PSM and extension of the tumor beyond the thyroid capsule^54^, prompting surgeons to weigh the possibility of impacting the functionality and quality of life of the patient against the aggressive surgical approaches that are more likely to completely clear tumor burden^55,56^.

NCCN^2^ recommends completion thyroidectomy for well-differentiated thyroid cancer with PSM (Table 2)^57^.

### PSM for cancers affecting males only

#### Prostate

Prostate had the highest PSM rate (21.03%) of any cancer in men (Table 1). Reported PSM incidence for radical prostatectomy varies widely (4% to greater than 48%)^58,59^. Yossepowitch *et al*. reported an average PSM rate in contemporary robot-assisted laparoscopic radical prostatectomy series of 15% (range 6.5–32%)^60^. Our multivariable analysis showed a notable prostate cancer PSM decrease between 2007–2012, which may be a result of more cancers being detected at earlier stages due to heightened PSA screening. Movement away from PSA screening since 2012 may reverse this trend.

Generally, urologic surgeons prioritize the preservation of as much of the neurovascular bundle as possible to prevent urinary incontinence and erectile dysfunction, thus precluding the execution of a wide surgical excision of periprostatic tissue and risking the occurrence of PSM^61^. Prostate cancer patients of advanced age were less likely to have PSM, indicating that most elderly patients likely underwent radical prostatectomy without nerve sparing, perhaps due to poorer pretreatment sexual function.

The clinical impact of PSM after radical prostatectomy has been studied extensively and there is a consensus that PSM is associated with a significantly increased risk of biochemical recurrence^62–64^. However, prior work has shown that neither single PSMs, nor multiple PSMs, post-radical prostatectomy were independent risk factors for metastases, castration-resistant prostate cancer, cancer-specific death, or all cause death in a cohort of patients who received early salvage radiotherapy upon biochemical recurrence^64^. While more aggressive tumor characteristics have been identified as strong determinants for PSM, margin status was not an independent prognostic factor for survival^65^.

Per NCCN^2^, radiation therapy, either as adjuvant or early salvage therapy is indicated for PSMs to reduce the risk of biochemical recurrence (Table 2). The rapid adoption of new technologies for more targeted radiation such as intensity-modulated radiation has contributed to the increasing costs of radiation for prostate cancer, which is increasing faster than the costs of care for cardiovascular and pulmonary conditions^66^.

### PSM for cancers affecting females only

#### Ovarian

Ovarian had the highest PSM rate among cancers affecting women (35.0%) and showed a significant decline over the study period (Table 1, Fig. 1A).

Patients with ovarian cancer are generally treated with a combination of surgery and adjuvant chemotherapy. As mentioned previously, PSM is not part of the treatment paradigm for ovarian cancers^2^, as the surgical approach is focused on cytoreduction. Patients with newly-diagnosed, advanced ovarian cancer should have maximal surgical debulking to achieve minimal residual disease (residual implants < 1 cm).

Secondary debulking surgeries are potentially beneficial for patients who have an isolated relapse after a lengthy disease-free interval^67^. Studies have demonstrated that the volume of post-operatively residual disease inversely correlates with survival^68–70^. Interestingly, we found that PSM rates decreased over the study period on univariable analysis. We hypothesize that this may be due to increased attention to the importance of reducing residual disease in cytoreductive surgeries^71^. However, this decrease was not noted in the multivariable logistic regression, likely due other confounding patient/tumor/institutional factors.

Ovarian had the highest PSM rate among cancers affecting women (35.0%). PSM for these cancers showed a significant decline over the study period on univariate, but not multivariate, analysis (Table 1, Fig. 1A). We hypothesize that this decline may be due to increased attention to the importance of reducing residual disease in cytoreductive surgeries^70^. However, the interpretation of PSM for this tumor type is unclear, and the implications of these ovarian NCDB data are limited. Other data sources with detailed cytoreductive information would contribute to the surgical literature in a more relevant way.

#### Uterine

Uterine had the lowest PSM rate among cancers affecting women (4.32%). Elderly women, and patients with high stage and grade of uterine cancer, had higher PSM rates. PSM rates significantly increased throughout the study period (Table 1). To our knowledge, population-level PSM rates have not been reported in uterine cancer. The 26th Annual Report of the International Federation of Gynecology and Obstetrics (FIGO) states that 83% of endometrial cancer patients are diagnosed and treated at early-stage (FIGO I and II)^72,73^. Surgery is the cornerstone of managing these early-stage patients.

The impact of PSM on clinical endpoints in endometrial cancer has been studied extensively. One study in Stage II endometrial cancer with extension into the cervix suggested that minimizing PSM at the cervical junction with an extended (radical) hysterectomy and removal of parametria should reduce LRR and possibly improve survival^74^. Additional work has shown an increased rate of local recurrence in patients undergoing extra-fascial hysterectomy as opposed to radical hysterectomy, presumably due to the reduction in positive surgical margins at the cervical junction^75^. Most recently, an NCDB study showed increased hazards for death among patients with PSMs^76^.

NCCN^2^ recommends adjuvant radiation^77^ for PSM after extrafascial hysterectomy in the case of invasive cervical component (Table 2).

### Limitations

This study is based on information from a national database, and lacks details about the criteria used to categorize margin status. There is no pathology information about how many millimeters the tumor was from the specimen edge. Additionally, the data set lacks information on number of resections or sequential procedures. Accordingly, the PSM prevalence reported here likely underestimates true PSM rates at the time of initial surgical resection.

## Conclusion

This work serves to define the magnitude of PSM as a surgical challenge in the most common solid cancers in the US. Treatment algorithms vary considerably depending on cancer site and stage, but surgical excision remains axiomatic. A PSM commonly translates into worse prognosis and additional burden to patients and the healthcare system by necessitating adjuvant therapies. Our findings may be helpful to prioritize efforts aimed at mitigating PSM, thereby optimizing value and improving patient outcomes.

## Electronic supplementary material

Supplementary Table 1
