# Supplementary material for: Positive Surgical Margins in the 10 Most Common Solid Cancers
Source: Sci Rep. 2018 Apr 9;8:5686. doi: 10.1038/s41598-018-23403-5 (PMC5890246; doi:10.1038/s41598-018-23403-5)
Supplement: Supplementary file 1 — Supplementary Table 1 [file 41598_2018_23403_MOESM1_ESM.doc]

**Title:**Positive Surgical Margins in the 10 Most Common Solid Cancers

**Short Title:** Solid Tumor Positive Surgical Margins

**Authors**Orosco, Ryan K MD ^1^, Tapia, Viridiana J MPH ^1^, Califano, Joseph A MD ^1,2,4^, Clary, Bryan MD ^2^, Cohen, Ezra EW MD ^4,5^, Kane, Christopher MD ^4,6^, Lippman, Scott M MD ^4,5^ Messer, Karen PhD ^4^, Molinolo, Alfredo MD ^4^, Murphy, James D MD ^7^, Pang, John MD,^1^ Sacco, Assuntina MD,^4,5^ Tringale, Kathryn R,^1^ Wallace, Anne MD,^2,4+^ Nguyen, Quyen T MD/PhD ^1,2,3,4^

1 Division of Otolaryngology, Head and Neck Surgery, University of California San Diego, CA, USA

2 Department of Surgery, University of California San Diego, CA USA

3 Department of Pharmacology, University of California San Diego, CA, USA

4 Moores Cancer Center, University of California San Diego, CA, USA

5 Department of Medicine, Division of Hematology-Oncology, University of California San Diego, CA USA

6 Department of Urology, University of California San Diego, CA, USA

7 Department of Radiation Medicine and Applied Sciences, San Diego, CA, USA

**Corresponding author:**

Quyen T. Nguyen, MD/PhD

Professor of Surgery

University of California, San Diego

9500 Gilman Drive, MC 0647

La Jolla, CA 92093

Fax: 858-534-5270

q1nguyen@ucsd.edu

**Supplemental Table 1**. Patient Demographics for the 6,495,889 patients (1998-2012) categorized by individual tumor site for whom surgical margin data was available.

|  |  | **Breast** | | | **Prostate** | **Bladder** | **Colon & Rectum** | **Thyroid** | **Oral Cavity** | **Lung & Bronchus** | **Kidney & Renal Pelvis** | **Uterine** | **Ovarian** | **TOTAL** |
| --- | --- | --- | --- | --- | --- | --- | --- | --- | --- | --- | --- | --- | --- | --- |
|  |  | **Lumpectomy** | **Mastectomy** | **Total** |  |  |  |  |  |  |  |  |  |  |
|  | Number of Surgical Cases (N) | 1,479,230 | 1,051,335 | 2,530,565 | 761,637 | 241,791 | 1,218,834 | 314,459 | 120,826 | 462,482 | 361,240 | 391,997 | 92,058 | **6,495,889** |
| **Patient Factors** | Gender |  |  |  |  |  |  |  |  |  |  |  |  |  |
|  | Men | 0.4 | 1.5 | 0.9 | 100.0 | 73.9 | 50.5 | 23.6 | 61.1 | 50.0 | 61.3 | N/A | N/A | 33.54 |
|  | Women | 99.6 | 98.5 | 99.1 | N/A | 26.1 | 49.5 | 76.4 | 38.9 | 50.0 | 38.7 | 100.0 | 100.0 | 66.46 |
|  | Race |  |  |  |  |  |  |  |  |  |  |  |  |  |
|  | White | 76.2 | 74.2 | 75.4 | 75.1 | 83.1 | 75.3 | 73.4 | 80.6 | 80.7 | 74.9 | 76.1 | 76.6 | 76.04 |
|  | Black/AA | 9.0 | 9.8 | 9.4 | 10.4 | 4.9 | 9.9 | 6.7 | 5.0 | 7.2 | 9.7 | 8.1 | 6.7 | 8.95 |
|  | Other | 3.2 | 3.6 | 3.4 | 2.5 | 1.9 | 3.1 | 5.4 | 3.0 | 2.2 | 2.5 | 3.3 | 3.9 | 3.15 |
|  | Latino/Hisp | 11.5 | 12.3 | 11.9 | 12.0 | 10.1 | 11.7 | 14.5 | 11.4 | 9.9 | 12.9 | 12.4 | 12.7 | 11.86 |
|  | Age |  |  |  |  |  |  |  |  |  |  |  |  |  |
|  | <40 | 4.6 | 8.2 | 6.1 | 0.2 | 1.4 | 2.9 | 29.5 | 5.6 | 1.3 | 5.4 | 3.9 | 9.0 | 5.28 |
|  | 41-80 | 87.6 | 83.5 | 85.9 | 98.9 | 79.4 | 78.6 | 68.3 | 81.8 | 91.6 | 87.9 | 88.8 | 85.0 | 85.56 |
|  | >81 | 7.9 | 8.3 | 8.1 | 0.9 | 19.2 | 18.4 | 2.2 | 12.7 | 7.1 | 6.7 | 7.2 | 6.1 | 9.16 |
|  | Income Quartile (per quartile)* |  |  |  |  |  |  |  |  |  |  |  |  |  |
|  | 1st Quartile | 13.7 | 16.4 | 14.8 | 13.8 | 15.6 | 17.8 | 13.3 | 17.6 | 18.0 | 17.5 | 16.4 | 15.2 | 15.76 |
|  | 2nd Quartile | 20.3 | 22.0 | 21.0 | 21.1 | 23.1 | 23.3 | 19.9 | 25.0 | 24.0 | 23.6 | 22.9 | 22.3 | 22.04 |
|  | 3rd Quartile | 26.0 | 25.9 | 26.0 | 26.5 | 26.2 | 26.0 | 26.1 | 26.9 | 26.1 | 26.5 | 26.4 | 26.5 | 26.16 |
|  | 4th Quartile | 37.7 | 33.4 | 35.9 | 36.4 | 32.3 | 30.1 | 38.6 | 28.0 | 29.2 | 30.1 | 32.0 | 33.4 | 33.68 |
|  | Charlson Index* |  |  |  |  |  |  |  |  |  |  |  |  |  |
|  | 0 | 62.1 | 58.6 | 60.7 | 61.8 | 52.0 | 48.2 | 68.7 | 55.2 | 38.1 | 54.0 | 55.8 | 82.5 | 56.47 |
|  | 1 | 7.5 | 9.2 | 8.2 | 10.5 | 14.0 | 14.1 | 10.4 | 11.2 | 24.1 | 16.8 | 14.8 | 14.4 | 12.05 |
|  | 2 | 1.4 | 2.1 | 1.7 | 1.4 | 4.4 | 4.8 | 2.0 | 3.0 | 8.7 | 5.5 | 3.3 | 3.1 | 3.21 |
|  | Insurance Status |  |  |  |  |  |  |  |  |  |  |  |  |  |
|  | Not Insured | 1.7 | 2.4 | 2.0 | 1.3 | 2.1 | 2.6 | 2.7 | 4.1 | 1.7 | 2.9 | 3.4 | 4.6 | 2.26 |
|  | Private Insurance | 56.0 | 54.6 | 55.5 | 62.9 | 31.8 | 36.5 | 71.2 | 40.6 | 34.2 | 46.8 | 50.7 | 52.9 | 50.07 |
|  | Medicaid | 3.8 | 5.4 | 4.5 | 1.5 | 2.6 | 3.4 | 5.0 | 5.9 | 3.8 | 4.3 | 4.2 | 5.7 | 3.86 |
|  | Medicare | 35.8 | 34.8 | 35.4 | 31.2 | 60.6 | 54.8 | 18.2 | 45.8 | 57.4 | 43.1 | 39.0 | 34.2 | 41.04 |
|  | Other Government | 0.7 | 0.8 | 0.8 | 1.0 | 0.6 | 0.6 | 1.0 | 1.0 | 0.8 | 0.9 | 0.8 | 0.9 | 0.80 |
|  | Unknown | 1.9 | 2.0 | 1.9 | 2.1 | 2.2 | 2.0 | 1.9 | 2.6 | 2.0 | 2.0 | 1.9 | 1.7 | 1.97 |

| **Tumor Factors** | T-Category* |  |  |  |  |  |  |  |  |  |  |  |  |  |
| --- | --- | --- | --- | --- | --- | --- | --- | --- | --- | --- | --- | --- | --- | --- |
|  | In Situ | 23.3 | 15.3 | 20.0 | 0.0 | 5.0 | 6.6 | 0.0 | 4.5 | 0.2 | 0.3 | 1.1 | N/A | 9.38 |
|  | T1 | 56.6 | 39.8 | 49.6 | 45.8 | 21.9 | 13.7 | 53.3 | 48.8 | 45.4 | 62.2 | 74.7 | 32.5 | 43.25 |
|  | T2 | 16.4 | 29.4 | 21.8 | 25.6 | 14.5 | 15.8 | 21.0 | 21.7 | 35.7 | 13.8 | 4.0 | 5.8 | 20.04 |
|  | T3 | 0.9 | 7.6 | 3.7 | 3.7 | 3.8 | 49.3 | 14.4 | 5.4 | 7.9 | 10.7 | 2.9 | 24.9 | 13.74 |
|  | T4 | 0.4 | 3.3 | 1.6 | 0.8 | 5.5 | 10.6 | 5.5 | 13.6 | 5.5 | 2.1 | 1.1 | N/A | 3.99 |
|  | Grade |  |  |  |  |  |  |  |  |  |  |  |  |  |
|  | Well Differentiated | 22.0 | 14.4 | 18.8 | 1.8 | 17.8 | 10.3 | 14.1 | 25.9 | 12.1 | 12.3 | 39.5 | 10.3 | 15.38 |
|  | Moderately Differentiated | 36.4 | 36.4 | 36.4 | 51.5 | 21.3 | 60.6 | 2.8 | 40.9 | 37.3 | 40.6 | 27.1 | 16.1 | 40.03 |
|  | Poorly Differentiated | 26.1 | 35.1 | 29.8 | 44.3 | 33.8 | 15.8 | 1.1 | 11.8 | 35.7 | 23.4 | 18.8 | 43.5 | 26.92 |
|  | Undifferentiated | 1.2 | 1.4 | 1.3 | 0.4 | 14.8 | 1.4 | 0.7 | 0.5 | 3.3 | 6.6 | 3.0 | 9.2 | 2.31 |
|  | Not Determined/Not Stated | 14.4 | 12.7 | 13.7 | 2.1 | 12.3 | 11.9 | 81.2 | 21.0 | 11.6 | 17.1 | 11.6 | 20.9 | 15.36 |
| **Healthcare System Factors** | Hospital Cancer Volume |  |  |  |  |  |  |  |  |  |  |  |  |  |
|  | <25^th^ Percentile | 24.7 | 26.1 | 25.3 | 25.3 | 26.0 | 25.1 | 26.8 | 50.2 | 25.8 | 25.4 | 25.1 | 23.1 | 49.49 |
|  | 25th to <75th Percentile | 50.0 | 49.4 | 49.7 | 49.7 | 48.0 | 49.8 | 48.0 | 28.3 | 49.0 | 49.3 | 49.7 | 48.1 | 25.39 |
|  | >= 75^th^ Percentile | 25.4 | 24.6 | 25.0 | 25.0 | 26.0 | 25.1 | 25.3 | 21.6 | 25.1 | 25.3 | 25.2 | 28.8 | 25.11 |
|  | Facility Type |  |  |  |  |  |  |  |  |  |  |  |  |  |
|  | Community Cancer Program | 11.2 | 11.3 | 11.2 | 7.1 | 13.7 | 14.3 | 7.3 | 8.5 | 8.6 | 8.5 | 7.2 | 5.4 | 10.50 |
|  | Comprehensive Community Cancer Program | 59.3 | 59.7 | 59.5 | 52.2 | 54.3 | 59.0 | 51.9 | 43.6 | 55.1 | 51.2 | 53.4 | 51.2 | 56.43 |
|  | Academic Cancer Program | 29.3 | 28.9 | 29.1 | 40.5 | 31.9 | 26.6 | 40.7 | 47.9 | 36.1 | 40.1 | 39.2 | 43.3 | 32.91 |
|  | Other | 0.2 | 0.2 | 0.2 | 0.2 | 0.1 | 0.1 | 0.1 | 0.0 | 0.2 | 0.2 | 0.2 | 0.1 | 0.16 |
|  | Geographic Region |  |  |  |  |  |  |  |  |  |  |  |  |  |
|  | East Coast | 46.7 | 41.1 | 44.4 | 40.1 | 45.2 | 43.8 | 45.2 | 40.3 | 45.1 | 41.6 | 43.5 | 41.5 | 43.57 |
|  | Central | 36.1 | 42.1 | 38.6 | 42.7 | 41.6 | 41.5 | 37.3 | 43.0 | 41.7 | 43.9 | 40.4 | 39.7 | 40.39 |
|  | West/Mountain | 36.1 | 16.8 | 17.0 | 17.2 | 13.2 | 14.7 | 17.5 | 16.6 | 13.3 | 14.5 | 16.1 | 18.8 | 16.04 |
